# New *N*-(oxazolylmethyl)-thiazolidinedione Active against *Candida albicans* Biofilm: Potential Als Proteins Inhibitors

**DOI:** 10.3390/molecules23102522

**Published:** 2018-10-02

**Authors:** Gabriel Marc, Cătălin Araniciu, Smaranda Dafina Oniga, Laurian Vlase, Adrian Pîrnău, Mihaela Duma, Luminița Măruțescu, Mariana Carmen Chifiriuc, Ovidiu Oniga

**Affiliations:** 1 Faculty of Pharmacy, “Iuliu Hațieganu” University of Medicine and Pharmacy, 8 Victor Babes Street, 400012 Cluj-Napoca, Romania; Marc.Gabriel@umfcluj.ro (G.M.); laurian.vlase@umfcluj.ro (L.V.); ooniga@umfcluj.ro (O.O.); 2 National Institute for Research and Development of Isotopic and Molecular Technologies, 67-103 Donat Street, 400293 Cluj-Napoca, Romania; adrian.pirnau@itim-cj.ro; 3 State Veterinary Laboratory for Animal Health and Safety, 1 Piata Marasti Street, 400609 Cluj-Napoca, Romania; duma.mihaelacj@yahoo.com; 4 Department of Microbiology, Faculty of Biology, University of Bucharest, 1-3 Portocalelor Street, 60101 Bucharest, Romania; lumi.marutescu@gmail.com (L.M.); carmen_balotescu@yahoo.com (M.C.C.); 5 Research Institute of the University of Bucharest-ICUB, 91-95 Independentei Street, 050095 Bucharest, Romania

**Keywords:** oxazole, thiazolidine-2,4-dione, biofilm, *Candida albicans*, adhesion, invasins, Als

## Abstract

*C. albicans* is the most frequently occurring fungal pathogen, and is becoming an increasing public health problem, especially in the context of increased microbial resistance. This opportunistic pathogen is characterized by a versatility explained mainly by its ability to form complex biofilm structures that lead to enhanced virulence and antibiotic resistance. In this context, a review of the known *C. albicans* biofilm formation inhibitors were performed and a new *N*-(oxazolylmethyl)-thiazolidinedione scaffold was constructed. 16 new compounds were synthesized and characterized in order to confirm their proposed structures. A general antimicrobial screening against Gram-positive and Gram-negative bacteria, as well as fungi, was performed and revealed that the compounds do not have direct antimicrobial activity. The anti-biofilm activity evaluation confirmed the compounds act as selective inhibitors of *C. albicans* biofilm formation. In an effort to substantiate this biologic profile, we used in silico investigations which suggest that the compounds could act by binding, and thus obstructing the functions of, the *C. albicans* Als surface proteins, especially Als1, Als3, Als5 and Als6. Considering the well documented role of Als1 and Als3 in biofilm formation, our new class of compounds that target these proteins could represent a new approach in *C. albicans* infection prevention and management.

## 1. Introduction

*Candida* spp. are normally commensals found in the gastrointestinal tract, genitourinary tract or oropharyngeal tract of healthy people, but can become opportunistic pathogens that cause superficial infections (oral or vaginal candidiasis), deep-seated infections or systemic infections. Candidiasis diagnosis have increased recently due to disproportionate use of broad spectrum antibiotics, use of immunosuppressive drugs, malnutrition, aging population and the amplified use of medical devices [1,2].

*C. albicans* is the most prevalent and problematic of all *Candida* species, as it is responsible for 50% of the cases of candidiasis and is the fourth most common cause of nosocomial infections in the USA [1].

The pathogenic potential of this microbial strain is explained by its ability to adapt to various habitats and to form surface-attached microbial communities (biofilms) [3]. Biofilm formation on tissues surfaces leads to superficial infections, while the presence of biofilm on inert substrates, such as medical devices, is directly linked with systemic infections [4,5,6,7,8]. Biofilm-forming ability is associated with persistent candidemia [8] and also with an increased risk of mortality in patients with *C. albicans* bloodstream infections [9]. Also, biofilm formation is a central element in the acquisition of fungal resistance [10,11].

In the human body, biofilm is rarely the product of a single microbial species, instead polymicrobial biofilms are frequently present. This microbial synergy, between *C. albicans* and bacteria, can lead to enhanced virulence, increased biofilm formation, increased pathogenicity and thus more severe infections, increased antimicrobial resistance and even increased mortality. Most frequently, dual-species biofilm formed between *C. albicans* and *Streptococcus mutans* or *Streptococcus gordonii* have been isolated from denture stomatitis, peritonitis, periodontitis and dental caries, while *C. albicans* and *S. aureus* dual-biofilms are associated with vaginal, oral or blood stream infections, as well as medical-devices related biofilms (artificial heart valves, vascular catheter). *C. albicans* can also form a dual-biofilm with *P. aeruginosa* (respiratory tract infections, wounds) or *E. faecalis*, *C. difficile* (gastrointestinal tract infections) [7,12,13,14]. 

*C. albicans* biofilm is a complex structure that incorporates round yeast cells (blastospores), pseudohyphal cells (ellipsoidal cells) and hyphal cells (chains of cylindrical cells), both of which are interspersed with a polymeric extracellular matrix (ECM), which covers and protects the cells [15]. Biofilm formation is initiated by the adherence of round yeast cells to the substrate (adherence/“seeding” step); this stage is essential for biofilm formation [12,13]. The next step (initiation step) is characterized by a rapid proliferation of the adhered yeast cells, which also produce early-stage filamentation (hyphae or and pseudohyphae) [13,15,16]. This is followed by an accumulation of extracellular matrix that incorporates the network of polymorphic cells and provides the biofilm with a structured appearance, protection from chemical and physical injury, as well as high-level drug resistance (maturation step) [3,14]. The final stage of biofilm formation is known as the “dispersal step” in which round yeast cells are released to seed new substrates [12,13,15].

The key molecules in *C. albicans* biofilm formation are members of the agglutinin-like sequence proteins family (Als) [13,15,17]. This family encompasses eight members (Als1 to Als7 and Als9) with varied degrees of structural and functional similarities [18,19]. Although most Als proteins have clear adhesion functions, their multiple roles are just now beginning to be discovered. Thus Als1, Als3 and Als5 are adhesins, with broad host substrate specificity, that can mediate adherence to endothelial cells, oral epithelial cells, gelatine, fibronectin, fibrinogen, type IV collagen, laminin and salivary pellicle [3,20,21,22]. A particular form of adherence is represented by biofilm formation, which seems to be the special characteristic of Als1 (responsible for the initial adherence step) and Als3 (mainly expressed in hyphae cells, responsible for initiation and maturation phases) [3,13,15,20,23]. Als3 is also accountable for binding other microbial strains (*S. gordonii*) and thus is key for the formation of co-infections and polymicrobial biofilms [12,13,20]. Als3 increases *C. albicans* virulence by acting as an invasin at the level of epithelial cells (key for oropharyngeal candidiasis) or the endothelial cells lining the vasculature (key for deep tissues infections) [16,20,24,25]. Host cell invasion can be achieved via 2 distinct mechanisms: Fungal-induced endocytosis (passive processes that uses Als3 as well as other invasins like the Ssa1, a member of the HSP70 family of heat shock proteins) and active penetration (uses Als3 in collaboration with hydrolytic enzymes) [24]. Moreover, Als3 is also responsible for *C. albicans* metabolic flexibility as it serves as a receptor for ferritin and thus mediates iron acquisition from the host [20]. 

Because of the increase in the *C. albicans* infections prevalence, as well as the increase in antifungal drug resistance, anti-biofilm therapeutic strategies have become sorely needed [11,26].

The search for efficient inhibitors of *Candida* biofilm identified a series of natural compounds that could interfere with various stages of the process including: caffeic acid derivatives [27], usnic acid (a lichen secondary metabolite) [28], various lichen extracts [29], plant essential oils [30,31], probiotic cells supernatant products [32], 5-hydroxymethyl-2-furaldehyde from marine bacterium [33], magnolol [34,35], dracorhodin from the exudates of the fruit of *Daemonorops draco* [36], shearinines D and E obtained from a *Penicillium* sp. isolate [37], and other phytocompounds [38]. However, most of these inhibitors are either mixtures of natural compounds or highly complex structures that are not easily obtainable in the laboratory.

In the series of small molecules with anti-biofilm activity we can include: Alizarin and chrysazin [39], miltefosin [40], filastatin [41], aliskiren [42], various phenylthiazole derivatives [43,44] and thiazole Schiff bases [45]. The most interesting compounds were those identified by screenings of large library of compounds; such as 9029936 and 7977044 discovered by Romo et al. [46] from a library of more than 30,000 compounds. A screening of more than 20,000 compounds performed by Pierce et al. [47] led to the identification of a diazaspiro-decane scaffold (compounds 61894700, 80527891, 95143226, 17159859).

**Figure 1:**
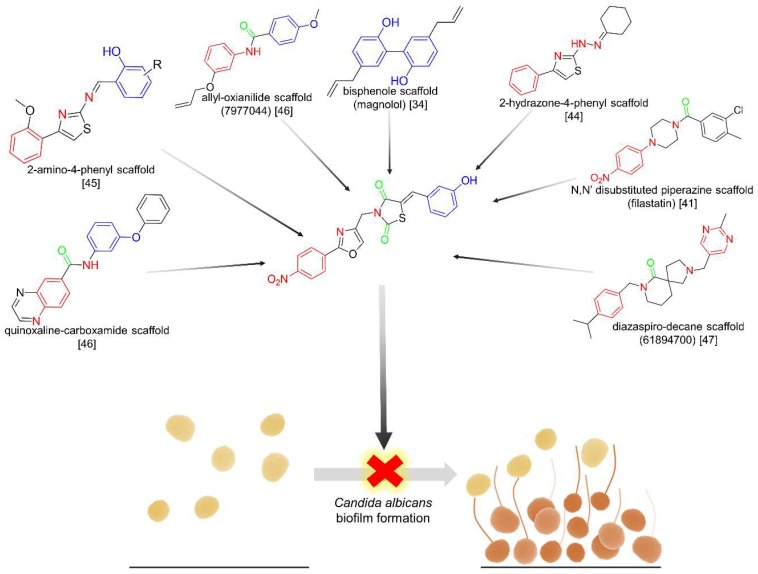
The new *N*-(oxazolylmethyl)-thiazolidinedione scaffold, encompassing various structural moieties present in known anti- biofilm agents.

Based on the structure of known *Candida* biofilm inhibitors, our research efforts focused on obtaining a new scaffold that encompasses various structural moieties contained in different active molecules, as shown in Figure 1.

Subsequently, we evaluated the general antimicrobial potential, as well as the general anti-biofilm activity. Our compounds proved to be selectively active against *Candida* biofilm formation with no effects against microbial cell viability or other microbial biofilm formation. In order to propose a possible mechanism for this biological activity, we also conducted a series of in silico determinations that suggest that our compounds act as Als inhibitors. 

## 2. Results and Discussion

### 2.1. Chemistry

A total of 16 new compounds (**6a**–**d**, **7a**–**d**, **8a**–**d**, **9a**–**d**) have been synthesized by the *N*-alkylation of various previously reported intermediates (**5a**–**d**) [48,49] with a series of 4-(chloromethyl)-2-phenyloxazoles (intermediate compounds **2a**–**d**).

**Figure 2:**
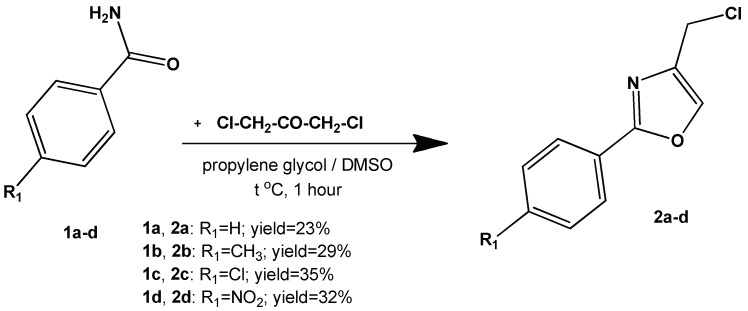
The synthesis of the 4-(chloromethyl)-2-phenyloxazoles intermediates (**2a**–**d**).

The 4-(chloromethyl)-2-phenyloxazoles (intermediate compounds **2a**–**d**) were obtained using the cyclisation of an amide with a α-haloketone to an oxazole, as shown in Figure 2. This method is based on the Blümlein-Lewy reaction, later reported by Bredereck and co-workers as “formamide synthesis” [50,51]. These intermediates were previously reported using a different synthetic protocol, a closed vial in hot oil bath [52,53]. Our modification of the technique confers the advantage of using the conventional reflux method under condenser, at atmospheric pressure, without catalysis. However, we admit the relatively low yields obtained (yields range = 23–35%) and the difficulty in product isolation.

The MS spectra of intermediates **2a**–**d** revealed the molecular ions, with a specific isotopic pattern due to ^35^Cl and ^37^Cl isotopes. The IR spectra showed the lack of a strong νC=O signal, characteristic for a primary amide, which confirms the successful cyclisation of the primary amide to oxazole. Other specific signals that confirm the formation of the oxazole ring are: A sharp signal with medium intensity, found between 3091 and 3148 cm^−1^ (corresponding to the stretching of the νC_5_-H) and the endocyclic νC=N bond, with a medium-strong signal between 1586 and 1593 cm^−1^. The aliphatic νC-Cl bond gives a strong signal between 690 and 702 cm^−1^. Signals that are specific to the intermediate compound **2d** are due to the nitro moiety that gave two characteristic signals caused by the asymmetric, respectively symmetric, stretching of the νN=O bond at 1522 and 1327 cm^−1^.

**Figure 3:**
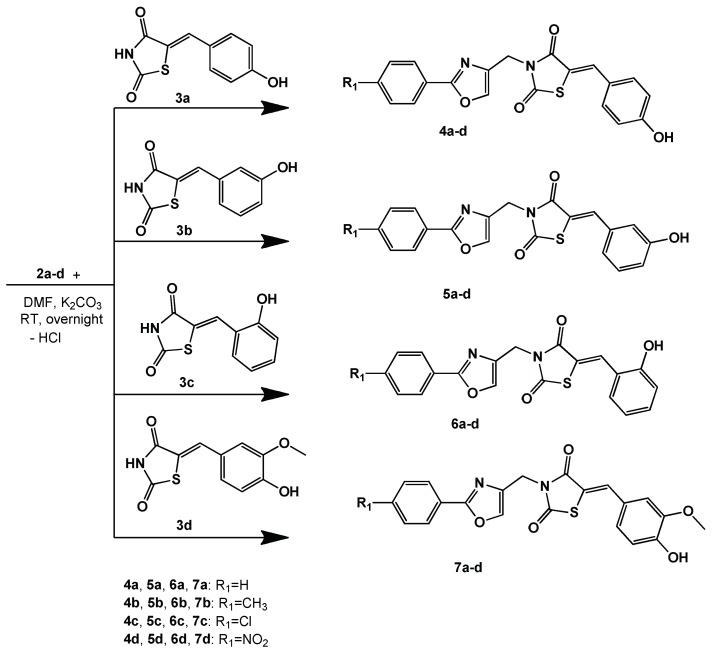
The synthesis of the *N*-(oxazolylmethyl)-thiazolidinedione (**4**,**5**,**6**,**7**: **a**–**d**).

The intermediates **2a**–**d** were used in alkylation reactions with previously described [48,49] thiazolidine-2,4-dione intermediates (**3a**–**d**), as can be observed in Figure 3. It is important to note that the alkylation reaction could have led to *O*-alkylation (like in the case of using chloroacetamide derivatives [54]) or *N*-alkylation (when non-amide substituents are used—Ph-CH_2_-Cl [55]). Our spectral data of the final compounds is consistent with the data proposed for *N*-alkylation structures.

The MS spectra of all final compounds confirmed the presence of the molecular ions.

By analyzing the IR spectra, we were able to identify a phenolic νO-H stretching, as a broad band between 3369–3523 cm^−1,^ that has led us to confirm that a *N*-alkylation took place, and not an *O*-alkylation. The presence of the oxazole ring was confirmed by a sharp signal between 3106–3166 cm^−1^ (νC_5_-H) and a strong signal between 1515–1521 cm^−1^ (νC=N). The thiazolidinedione was characterized by 2 νC=O, in two groups, shown as strong signals at 1755–1717 cm^−1^ and 1664–1693 cm^−1^. Specific signals were also identified corresponding to the NO_2_ contain compounds (2 bands due to νN=O asymmetric and symmetric stretching between 1521–1512 cm^−1^, respectively 1342–1336 cm^−1^) and the ether vanillin derivatives (νC-O-C ether bond appeared as strong signal between 1239–1284 cm^−1^).

The *N*-alkylation is also supported by ^1^H-NMR data. As such, we can observe the lack of a broad signal from a very deshielded proton (N-H from thiazolidinedione) at >12 ppm, and the presence of a broad signal corresponding to the OH proton between 9.90–10.67 ppm. ^13^C-NMR is also consistent with the proposed structures, as it shows 2 groups of C=O signals between 167.11–167.91 ppm and 165.87–165.06 ppm (corresponding to the thiazolidinedione), 3 carbon atoms from oxazole (C_2_: 160.01–160.86 ppm, C_4_: 138.54–137.98 ppm, C_5_: 136.61–135.79 ppm), and a -CH_2_- bridge between 37.72–37.53 ppm.

### 2.2. Biological Assays

#### 2.2.1. Antimicrobial Activity—Initial In Vitro Qualitative Screening Study

Antimicrobial activity was evaluated using a series of Gram-positive strains, Gram-negative strains and a fungal strain. This determination aimed at establishing the biological profile of the newly synthesized compounds, namely their antimicrobial potential. Results of the initial in vitro qualitative screening are shown in Table 1. An overall analysis reveals that all compounds have a degree of antimicrobial activity, but it is very low compared with standard antimicrobial agents used as controls.

The best antimicrobial activity was obtained against *C. albicans* by compounds **4a**, **4c**, **6a**, **7a** that had an inhibition of growth zone diameter of 16 mm. However, this action is mediocre compared with the fluconazole standard (24 mm).

Considering antibacterial activity, the compounds seem to be more active against Gram-negative strains compared with Gram-positive strains. The most significantly active compounds were **7c** and **7d,** against the *E. coli* strain. A mediocre effect was observed also against *E. faecalis*, while most compounds had a negligible activity against *S. aureus*.

#### 2.2.2. Antimicrobial Activity—In Vitro Quantitative Assay

The quantitative assay was performed in order to more precisely evaluate the direct antimicrobial effects, as initial screenings determined a potentially moderate antimicrobial effect. The results from these investigations, shown in Table 2, clearly demonstrate that the newly synthesized compounds do not have relevant direct antimicrobial effect at the small concentrations that can be achieved at cellular levels during antibiotic therapy.

Being deprived of direct antimicrobial effects, these compounds do not create an increase in bacterial resistance by increasing the selection pressure via bacteriostatic or bactericidal effects. This could be viewed as a positive feature, if these compounds possess anti-biofilm effects, as initially assumed when designing the scaffold. Also, by specifically targeting *C. albicans* biofilm, the new agents could be used without risk of causing imbalances of the commensal flora.

#### 2.2.3. Anti-Biofilm Activity Assay

The crystal violet staining method provides a total quantification of biofilm biomass, as it includes various cells and extracellular matrix [7].

Anti-biofilm activity can be independent of the direct antimicrobial activity; as a consequence, new compounds must be evaluated in parallel for both properties.

Our anti-biofilm screening results, presented in Table 3, indicate that the tested compounds were mainly active against *C. albicans* biofilm formation. Out of a total of 16 compounds, 14 of them were active against biofilm formation at minimal biofilm eradication concentrations (MBEC), smaller than the standard used (berberine). The most active compound was **5d**, which still maintained anti-biofilm effects even at concentrations as low as 0.038 mg/mL. 10 of the compounds were active at concentrations (0.078 mg/mL) four times smaller than the standard.

Activity against the biofilm of Gram-negative strains appears to be manifested only at high concentrations, or absent. Concerning Gram-positive strains, some of the compounds (**6b**, **6c** and **7c**) seem to be moderately active against *E. faecalis* biofilm formation with MBEC values equal to that of the standard. 

An optimal anti-biofilm agent has to be active at small concentrations, without exercising positive selection pressure via direct antimicrobial effect. In the same time, considering the fact that in vivo biofilm is usually polymicrobial, it could be beneficial for a new drug-candidate molecule to have effect on multiple microbial strains. However, our results clearly indicate that the tested compounds are active predominantly against *C. albicans* biofilm, which is in agreement with our research hypothesis of obtaining a new scaffold of molecules that target this fungal strain.

### 2.3. In Silico Studies

Given the biological evaluation results, we aimed at identifying a potential mechanism of action for our compounds. Due to the specificity against *C. albicans* biofilm formation, we investigated the affinity that our molecules could have against the Als family proteins, which are known to be key elements in *Candida* spp. adhesion, biofilm formation and virulence. Direct binding of the investigated compounds to the Als surface proteins could render them unavailable for key interactions that mediate their biological effects. 

#### 2.3.1. Molecular Docking Study

The Als proteins are structurally related, all having a basic structure formed by: A N terminus signal peptide, a 300-amino-acids immunoglobulin-like domain, a threonine-rich domain, a central domain made up of variable number of 36-amino-acid tandem repeats, a heavily glycosilated serine and threonine rich domain, and a glycosylphosphatidylinositol anchorage sequence that is cleaved in order to ensure the covalent binding of the protein to the cell wall [20]. The hydrophobic central domain seems to be involved in adherence by binding to some substrates like polystyrene [56].

Als3, which is a well-documented invasin, seem to be able to interact with specific receptors on the surface of host cells: E-cadherin on epithelial cells and *N*-cadherin on endothelial cells. These interactions presumably take place via the immunoglobulin-like domain of Als3 [3,20,24].

The tested compounds were docked into the binding sites of Als surface proteins of *C. albicans*. The predicted best binding affinity of the conformation of each compound to the binding site of the surface protein are presented in Table 4.

In order to better asses the influence of the different substituents located on the phenyl-oxazole vs. those located on the benzylidene moiety, we compared the averages of the binding energies and calculated the standard deviation, as shown in Table 5.

When interpreting these results, we considered that a higher standard deviation for the binding affinity shows that the variation of substituent induces bigger differences in binding mode, while a reduced standard deviation translates by a decreased influence of the substituent on the binding affinity. As such, we can observe that the substituents on the 2-phenyloxazole residue (H, 4-CH_3_, 4-Cl, 4-NO_2_) tend to have a lesser impact on binding than those from the benzylidene moiety. The position of the OH group on the benzylidene seems to have the highest influence on binding affinity: Optimal affinity is achieved by inserting the OH in the 3rd or 4th position, while the insertion of an extra methoxy group is unfavorable.

By analyzing the predicted inhibition constants, shown in Table 6, and also the binding affinities, it is apparent that all tested compounds tend to have a better binding potential than that of the berberine sulphate standard.

All compounds, except **6d**, have a good inhibition potential against Als1. This could explain the biological activity, as it is well documented that Als1 is key for *C. albicans* adherence and controls the initial “seeding” step leading to biofilm production. Also, together with Als3, Als1 modulates the initiation step and maturation step of biofilm development.

Another noticeable feature is represented by the very good inhibition potential of all compounds against Als5 and Als6. Although Als6’s roles are not yet fully understood, Als5 is proved to be a key adhesin together with Als1 and Als3.

When considering the potential to inhibit Als3, the most significant Als target, results showed that all compounds are significantly superior to the standard berberine, and 5 compounds have a Ki <20 nM.

*Candida* biofilm formation is a complex process that involves several regulators that act as transcription factors (Bcr1, Efg1), as well as multiple adhesins (Als, Hwp1, Eap1, PGA10) and other factors. It is important to note that these molecules perform complementary functions and work together for biofilm formation [3,15,21]. As a result, direct inhibition of one of these factors does not necessarily translate into good biologic activity, as compensatory mechanisms can be activated. From this perspective, compounds that act simultaneously as inhibitors of more targets are preferred. This could also explain the lack of a direct causality between the potential inhibition of Als and results obtained in the direct biological anti-biofilm determinations.

As such, the most interesting compounds from our series seem to be **4b** and **5d**, which have good inhibition potential against 6 of the 9 Als targets, and especially against Als3, that is believed to be the most important Als protein for biofilm development and a key element of overall Candida albicans virulence.

#### 2.3.2. Docking Mode of the Compounds to the Binding Site of Als

**Figure 4:**
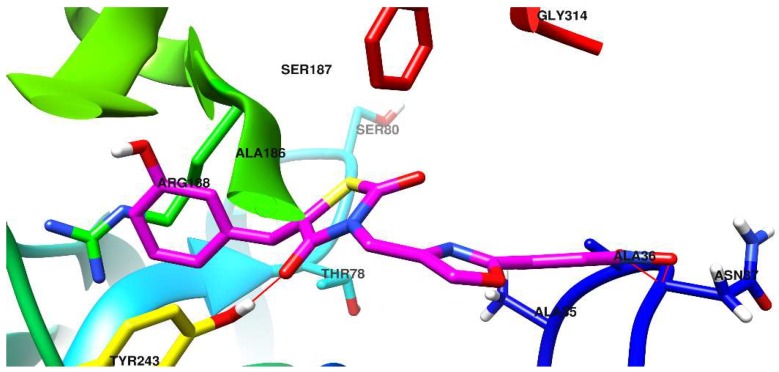
Compound **5d** docked in the binding site of the *C. albicans* Als1 surface protein.

**Figure 5:**
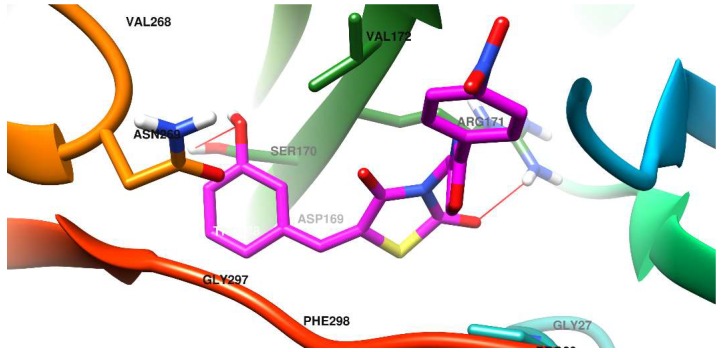
Compound **5d** docked in the binding site of the *C. albicans* Als3 surface protein.

The docking pose of one of the most promising new compounds, **5d**, against Als1 and Als3 is shown in Figure 4 and Figure 5.

The binding pocket of Als1 is a rather flattened surface, shaped as 2 adjacent Y. In other words, each of its’ distal and proximal extremities present 2 sub-pockets formed by protruding moieties that subdivide the central binding site.

The distal extremity is characterized by a protruding moiety made up by the vicinal joint of Tyr243 and Ala186. As a result, 2 distal sub-pockets are formed: A small pocket with very limited use (non-druggable) and a larger, predominantly lipophilic one, which accommodates the phenol ring of compound **5d**. The gateway to the large distal sub-pocket is an Arg168 residue that interacts with the π electrons from the phenol aromatic ring, via the guanidine moiety.

The thiazolidinedione ring of compound **5d** is situated in the central part of the binding pocket, away from residues capable of interaction, with the notable exception of a hydrogen bond formed between the Tyr243 phenol OH group and a C=O group from the thiazolidinedione.

The proximal extremity of the Als1 binding site also presents a protruding moiety formed by the spatial proximity of the C-terminal end (Gly314-Tyr315) and a loop (Ala35-Ala36-Asn37). The 2 resulting proximal sub-pockets are mostly lipophilic, but each has at least one hydrophilic residue: The small sub-pocket has Tyr35, while the more voluminous sub-pocket has Asn37.

The oxazolyl-phenyl structure from **5d** acts primarily as a spacer, with the two rings being co-planar, and forming a large entity that cannot fit in the small proximal sub-pocket. However, this makes it capable of favoring the spatial orientation of the nitro group in the vicinity of the peptide bond Ala36-Asn37, found in the larger proximal sub-pocket. Also, the methylene moiety from **5d** acts as a hinge and helps to direct the nitro-phenyl residue towards the more voluminous sub-pocket. As a result, polar interactions take place between the NO from the nitro group and the peptide bond Ala36-Asn37.

In the case of Als3, the predicted binding site is located in the core of the protein. It is shaped like a channel running across the protein. Access to this area is controlled by 2 entities: A beta-strand formed by Leu293-Arg294-Trp295-Thr296 and then by Gly297-Phe298-Arg299, that border and provide lining to the pocket and an omega loop Val268-Asn269-Ser270 that partially obstruct the binding site entrance. 

The OH phenol group from compound **5d** is conveniently oriented towards Ser170, with whom it forms a hydrogen bond, but also near Tyr271, thus allowing for supplementary interaction. The thiazolidine ring of **5d** sits in a polar region and interacts via a hydrogen bond formed between a C=O group and the Arg171 residue. The p-NO_2-_phenyl-oxazole form **5d** lingers towards the exit of the binding pocket, and because of the widening of the binding site, the nitro group does not manage to form reliable interactions.

#### 2.3.3. Analysis of Als Proteins Structure

**Figure 6:**
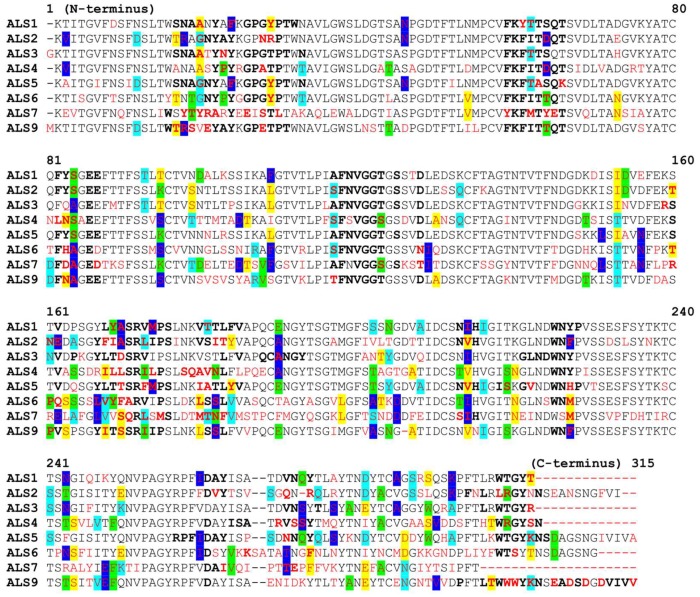
Sequence alignment for the Als targets.

A phylogenetic analysis of Als proteins reveals that these surface proteins are related, which was to be expected, as they are part of the same superfamily [18]. However, significant differences could be found between them. Figure 6 shows the sequence alignment for the Als targets, with amino acids found near the binding site marked in bold. After comparing the amino acid sequences, for every amino acid position in the studied Als proteins, the “normal” amino acid was considered the one which was identical in most proteins in that particular position. If the amino acid suffered a mutation and was different, it was depicted in red. If a mutation of an amino acid in a specific position is found in more than one protein, the letter corresponding to that mutation was highlighted with the same color, in all chains where it appeared.

The degree of similarity was assessed by calculating a similarity matrix, presented in Table 7. Result showed a high degree of similarity between Als1, Als3 and Als5 (>80%).

**Figure 7:**
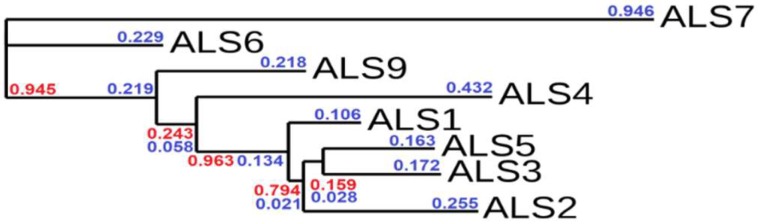
Sequence homology of Als proteins. The branch support values are depicted in red, while the branch length values are depicted in blue.

To better understand the degree of similarity, a phylogenetic tree was generated using phylogeny.fr [57], and is depicted in Figure 7. The length of the branches (blue) is proportional to the number of substitutions per site, while branch support (red) indicates the degree of similarity, as it characterizes common evolutionary background.

Both the similarity matrix and the phylogenetic tree indicate that, despite the fact that studied ALS sequences have a common ancestry, ALS7 has a different evolutionary path to the other ALS proteins. Also, Als6 seem to have evolved separately, whereas Als9 and Als1-5 share a common evolutionary ancestor. As in the case of the similarity matrix, the phylogenetic tree underlines the close connections between Als1, Als3 and Als5 which is also supported by the known biologic roles of these proteins (they all share a common adhesin function).

A comparative analysis of the active sites’ binding pockets from different Als, shown in Table 8, revealed that Als1, Als3 and Als5 are the proteins with the biggest volume and widest surface from the series considered. This suggests that they would be able to better accommodate larger ligands in their active sites. Also, these proteins have the lowest hydrophobicity ratios, which could indicate their tendency to form polar interactions at the level of the binding pockets. Als1, Als3, Als5 and Als6 have the highest percentage of polar amino acids, which could account for their ability for polar interactions with various ligands, including our *N*-(oxazolylmethyl)-thiazolidinediones (which contain various polar substituents: NO_2_, OH, CO).

## 3. Materials and Methods

### 3.1. General Information

All chemicals were of analytical reagent grade purity, and have been purchased from Merck (Darmstadt, Germany) or Sigma-Aldrich (Taufkirchen, Germany).

The uncorrected melting points were obtained by the open glass capillary method, using a MPM-H1 melting point apparatus (Schorpp Gerätetechnik, Überlingen, Germany).

MS spectra were obtained by using an Agilent 1100 series, in positive ionization with an Agilent Ion Trap SL mass spectrometer (70 eV) instrument (Agilent Technologies, Santa Clara, CA, USA). IR spectra were recorded after compression of the samples in KBr pellets, under vacuum, using a FT/IR 6100 spectrometer (Jasco, Cremella, Italy). The device was controlled using the computer interface software Spectra Manager. Assignment of IR signals was made using Know It All 7.8 by Bio-Rad Laboratories (Hercules, CA, USA).

The ^1^H-NMR and ^13^C-NMR were recorded on an Avance NMR spectrometer (Bruker, Karlsruhe, Germany) using DMSO-*d*_6_ as solvent. Chemical shift values are reported in δ units, relative to TMS as internal standard. All spectral data were in accordance with the proposed chemical structures.

Elemental analysis was performed by Vario El CHNS analyzer (Hanau, Germany). The results obtained for all synthesized compounds were in agreement with the calculated values.

### 3.2. Chemistry

General Procedure for the synthesis of the 4-(chloromethyl)-2-aryloxazoles (**2a**–**d**). 10 mmol of benzamides **1a**–**d** and 10 mmol 1,3-dichloroacetone were mixed well in a round bottom flask with 5 mL of propylene glycol and 0.5 mL of dimethylsulfoxide. Reactions were performed in an open vessel, under condenser, with vigorous magnetic stirring. Between the reaction flask and the condenser, a valve was designed from a small inner diameter conical glass adaptor, equipped with a small glass ball that moved vertically freely within, in order to reduce the volatilization of dichloroacetone. The mixture was refluxed for one hour. Upon completion of the reaction, the mixture was cooled to room temperature, 5 mL of methanol was added and the mixture was stirred well. Further, water was added carefully dropwise, in order to obtain a precipitate. The resulted solid was filtered under vacuum. The impure 4-(chloromethyl)-2-aryloxazoles **2a**–**d** were recrystallized twice from methanol-water and activated a charcoal mixture to give the appropriate pure **2a**–**d** intermediate products.

*4-(Chloromethyl)-2-phenyloxazole* (**2a**): white solid; mp = 55 °C (lit. 55.5–56 °C [52]); yield = 23%; FT IR (KBr) ν_max_ cm^−1^: 3091 (C_5_-H oxazole), 1593 (C=N), 702 (C-Cl); MS: *m*/*z* = 194.1 and 196.3 (M + 1 and M + 3 due to ^35^Cl and ^37^Cl); ^1^H NMR (DMSO-d_6_, 500 MHz) δ: 8.23 (s, 1H, oxazole C_5_-H), 7.94 (d, *J* = 7.9 Hz, 2H, Ar), 7.51–7.49 (m, 3H, Ar), 4.31 (s, 2H, -CH_2_-); ^13^C NMR (DMSO-d_6_, 125 MHz) δ: 161.27 (oxazole C_2_), 138.02 (oxazole C_4_), 136.58 (oxazole C_5_), 130.74, 129.55, 127.01, 126.39 (4 aromatic carbons), 41.13 (-CH_2_-).

*4-(Chloromethyl)-2-(p-tolyl)oxazole* (**2b**): white solid; mp = 94–95 °C (lit. 94–95 °C [52]); yield = 29%; FT IR (KBr) ν_max_ cm^−1^: 3128 (C_5_-H oxazole), 1587 (C=N), 693 (C-Cl); MS: *m*/*z* = 208.1 and 210.3 (M + 1 and M + 3 due to ^35^Cl and ^37^Cl); ^1^H NMR (DMSO-d_6_, 500 MHz) δ: 8.29 (s, 1H, oxazole C_5_-H), 7.71 (d, *J* = 8.1 Hz, 2H, Ar), 7.29 (d, *J* = 8.1 Hz, 2H, Ar), 4.38 (s, 2H, -CH_2_-), 2.59 (s, 3H, CH_3_-); ^13^C NMR (DMSO-d_6_, 125 MHz) δ: 160.59 (oxazole C_2_), 138.21 (oxazole C_4_), 135.73 (oxazole C_5_), 140.98, 130.65, 122.73, 120.84 (4 aromatic carbons), 42.01 (-CH_2_-).

*4-(Chloromethyl)-2-(4-chlorophenyl)oxazole* (**2c**): white solid; mp = 97 °C (lit. 97–98 °C [52]); yield = 35%; FT IR (KBr) ν_max_ cm^−1^: 3128 (C_5_-H oxazole), 1586 (C=N), 690 (C-Cl); MS: *m*/*z* = 228, 230, 232 (M + 1 and M + 3 due to ^35^Cl and ^37^Cl); ^1^H NMR (DMSO-d_6_, 500 MHz) δ: 8.24 (s, 1H, oxazole C_5_-H), 7.92 (d, *J* = 8.7 Hz, 2H, Ar), 7.49 (d, *J* = 8.7 Hz, 2H, Ar), 4.39 (s, 2H, -CH_2_-); ^13^C NMR (DMSO-d_6_, 125 MHz) δ: 160.44 (oxazole C_2_), 138.20 (oxazole C_4_), 135.91 (oxazole C_5_), 136.89, 129.49, 128.31, 124.42 (4 aromatic carbons), 41.63 (-CH_2_-).

*4-(Chloromethyl)-2-(4-nitrophenyl)oxazole* (**2d**): brown-yellow solid; mp = 138–139 °C (lit. 140 °C [53]); yield = 32%; FT IR (KBr) ν_max_ cm^−1^: 3148 (C_5_-H oxazole), 1587 (C=N), 1522, 1337 (N=O nitro), 702 (C-Cl); MS: *m*/*z* = 239.1 and 239.4 (M + 1 and M + 3 due to ^35^Cl and ^37^Cl); ^1^H NMR (DMSO-d_6_, 500 MHz) δ: 8.23 (s, 1H, oxazole C_5_-H), 8.12 (d, *J* = 8.6 Hz, 2H, Ar), 8.05 (d, *J* = 8.6 Hz, 2H, Ar), 4.32 (s, 2H, -CH_2_-); ^13^C NMR (DMSO-d_6_, 125 MHz) δ: 160.29 (oxazole C_2_), 138.27 (oxazole C_4_), 135.99 (oxazole C_5_), 145.91, 128.52, 126.58, 124.22 (4 aromatic carbons), 41.55 (-CH_2_-).

General Procedure for the Synthesis of the Intermediate Compounds **3a**–**d** (Z isomers) was based on a Knoevenagel condensation in alkaline medium, provided by the anhydrous sodium acetate. The condensation between the corresponding phenolic aldehydes and thiazolidine-2,4-dione was made under microwave irradiation in acetic acid. The synthetic protocol and the characterization of the intermediate compounds **3a**–**d** were previously reported [48,49].

General Procedure for the Synthesis of the Final Compounds (**4a**–**d**, **5a**–**d**, **6a**–**d** and **7a**–**d**). 5-(hydroxybenzylidene)-thiazolidine-2,4-diones intermediates (**3a**–**d**) were selectively alkylated on the nitrogen atom from the thiazolidine-2,4-dione ring, in alkaline medium using a modified protocol [58]. Over 1.05 mmol of intermediate compound **3a**–**d** and 1 mmol of intermediate compound **2a**–**d**, dimethylformamide (DMF) was added dropwise until their dissolution, in order to obtain the highest possible concentration of the intermediate compounds, to ensure a high reaction rate. After that, 2 mmol of anhydrous potassium carbonate and 1 mmol of anhydrous potassium iodide were added to this solution. The mixture was stirred overnight at room temperature. Upon completion of the reaction, the mixture was poured over ice cold saturated brine. A 10% sulfuric acid solution was added dropwise until complete precipitation of the product had occurred. The resulted solid was filtered under vacuum and dried. The remaining residue was crystallized twice from acetone, giving the pure final compounds **4a**–**d**, **5a**–**d**, **6a**–**d** and **7a**–**d**.

*(Z)-5-(4-Hydroxybenzylidene)-3-((2-phenyloxazol-4-yl)methyl)thiazolidine-2,4-dione* (**4a**): C_20_H_14_N_2_O_4_S, calcd: C 63.48%, H 3.73%, N 7.40%, S 8.47%, found: C 63.45%, H 3.74%, N 7.44%, S 8.46%; yellow solid; mp = 223–224 °C; yield = 57%; FT IR (KBr) ν_max_ cm^−1^: 3410 (O-H), 3145 (C_5_-H oxazole), 1755 (C=O), 1675 (C=O), 1590 (C=N); MS: *m*/*z* = 379.3 (M + 1); ^1^H NMR (DMSO-d_6_, 500 MHz) δ: 10.43 (br, 1H, OH), 8.25 (s, 1H, oxazole C_5_-H), 7.92 (d, *J* = 7.9 Hz, 2H, Ar), 7.88 (s, 1H, -CH=), 7.58 (d, *J* = 8.2 Hz, 2H, Ar), 7.52–7.50 (m, 3H, Ar), 6.93 (d, *J* = 8.2 Hz, 2H, Ar), 4.82 (s, 2H, -CH_2_-); ^13^C NMR (DMSO-d_6_, 125 MHz) δ: 167.19 (C=O), 165.11 (C=O), 161.29 (oxazole C_2_), 159.38 (ArC-OH), 138.01 (oxazole C_4_), 136.55 (oxazole C_5_), 134.57 (-CH=), 133.29, 130.99, 129.63, 127.05, 126.41, 125.61, 115.82 (7 aromatic carbons), 116.22 (TZD C_5_=), 37.61 (-CH_2_-).

*(Z)-5-(4-Hydroxybenzylidene)-3-((2-(p-tolyl)oxazol-4-yl)methyl)thiazolidine-2,4-dione* (**4b**): C_21_H_16_N_2_O_4_S, calcd: C 64.27%, H 4.11%, N 7.14%, S 8.17%, found: C 64.21%, H 4.01%, N 7.09%, S 8.05%; yellow solid; mp = 229–230 °C; yield = 53%; FT IR (KBr) ν_max_ cm^−1^: 3403 (O-H), 3140 (C_5_-H oxazole), 1737 (C=O), 1675 (C=O), 1593 (C=N); MS: *m*/*z* = 393.1 (M + 1); ^1^H NMR (DMSO-d_6_, 500 MHz) δ: 10.28 (br, 1H, OH), 8.28 (s, 1H, oxazole C_5_-H), 7.94 (s, 1H, -CH=), 7.75 (d, *J* = 8.0 Hz, 2H, Ar), 7.51 (d, *J* = 8.4 Hz, 2H, Ar), 7.28 (d, *J* = 8.0 Hz, 2H, Ar), 6.96 (d, *J* = 8.4 Hz, 2H, Ar), 4.80 (s, 2H, -CH_2_-), 2.61 (s, 3H, CH_3_-Ar); ^13^C NMR (DMSO-d_6_, 125 MHz) δ: 167.24 (C=O), 165.29 (C=O), 160.26 (oxazole C_2_), 160.23 (ArC-OH), 138.16 (oxazole C_4_), 135.90 (oxazole C_5_), 134.41 (-CH=), 140.11, 133.47, 130.84, 126.54, 125.69, 122.80, 116.81 (7 aromatic carbons), 116.32 (TZD C_5_=), 37.55 (-CH_2_-), 23.48 (-CH_3_).

*(Z)-3-((2-(4-Chlorophenyl)oxazol-4-yl)methyl)-5-(4-hydroxybenzylidene)thiazolidine-2,4-dione* (**4c**): C_20_H_13_ClN_2_O_4_S, calcd: C 58.18%, H 3.17%, N 6.79%, S 7.77%, found: C 58.21%, H 3.19%, N 6.82%, S 7.80%; yellow solid; mp = 236 °C; yield = 62%; FT IR (KBr) ν_max_ cm^−1^: 3410 (O-H), 3150 (C_5_-H oxazole), 1739 (C=O), 1685 (C=O), 1595 (C=N); MS: *m*/*z* = 413.2 and 415.5 (M + 1; ^35^Cl and ^37^Cl approx. 3:1 ratio); ^1^H NMR (DMSO-d_6_, 500 MHz) δ: 10.40 (br, 1H, OH), 8.25 (s, 1H, oxazole C_5_-H), 7.94 (d, *J* = 8.0 Hz, 2H, Ar), 7.89 (s, 1H, -CH=), 7.58 (d, *J* = 8.5 Hz, 2H, Ar), 7.50 (d, *J* = 8.0 Hz, 2H, Ar), 6.94 (d, *J* = 8.5 Hz, 2H, Ar), 4.82 (s, 2H, -CH_2_-); ^13^C NMR (DMSO-d_6_, 125 MHz) δ: 167.58 (C=O), 165.81 (C=O), 160.86 (oxazole C_2_), 160.37 (ArC-OH), 138.28 (oxazole C_4_), 135.93 (oxazole C_5_), 134.35 (-CH=), 136.82, 133.15, 129.79, 128.23, 125.86, 124.20, 116.94 (7 aromatic carbons), 116.82 (TZD C_5_=), 37.53 (-CH_2_-).

*(Z)-5-(4-hydroxybenzylidene)-3-((2-(4-nitrophenyl)oxazol-4-yl)methyl)thiazolidine-2,4-dione* (**4d**): C_20_H_13_N_3_O_6_S, calcd: C 56.73%, H 3.09%, N 9.92%, S 7.57%, found: C 56.70%, H 3.07%, N 9.91%, S 7.55%; yellow solid; mp = 257 °C; yield = 64%; FT IR (KBr) ν_max_ cm^−1^: 3504 (O-H), 3166 (C_5_-H oxazole), 1717 (C=O), 1664 (C=O), 1600 (C=N), 1521, 1336 (N=O); MS: *m*/*z* = 424.5 (M + 1); ^1^H NMR (DMSO-d_6_, 500 MHz) δ: 10.47 (br, 1H, OH), 8.25 (s, 1H, oxazole C_5_-H), 8.17 (d, *J* = 8.4 Hz, 2H, Ar), 8.09 (d, *J* = 8.4 Hz, 2H, Ar), 7.88 (s, 1H, -CH=), 7.59 (d, *J* = 8.2 Hz, 2H, Ar), 6.93 (d, *J* = 8.2 Hz, 2H, Ar), 4.83 (s, 2H, -CH_2_-); ^13^C NMR (DMSO-d_6_, 125 MHz) δ: 167.39 (C=O), 165.18 (C=O), 160.22 (oxazole C_2_), 160.55 (ArC-OH), 138.54 (oxazole C_4_), 135.99 (oxazole C_5_), 134.26 (-CH=), 145.10, 133.54, 128.35, 126.72, 125.73, 124.16, 117.09 (7 aromatic carbons), 116.35 (TZD C_5_=), 37.33 (-CH_2_-).

*(Z)-5-(3-Hydroxybenzylidene)-3-((2-phenyloxazol-4-yl)methyl)thiazolidine-2,4-dione* (**5a**): C_20_H_14_N_2_O_4_S, calcd: C 63.48%, H 3.73%, N 7.40%, S 8.47%, found: C 63.46%, H 3.74%, N 7.43%, S 8.45%; white solid; mp = 217 °C; yield = 74%; FT IR (KBr) ν_max_ cm^−1^: 3460 (O-H), 3143 (C_5_-H oxazole), 1734 (C=O), 1670 (C=O), 1599 (C=N); MS: *m*/*z* = 379.4 (M + 1); ^1^H NMR (DMSO-d_6_, 500 MHz) δ: 9.90 (br, 1H, OH), 8.23 (s, 1H, oxazole C_5_-H), 7.89 (s, 1H, -CH=), 7.96 (d, *J* = 8.0 Hz, 2H, Ar), 7.54–7.51 (m, 3H, Ar), 7.34 (t, *J* = 8.0 Hz, 1H, Ar), 7.09 (d, *J* = 8.0 Hz, 1H, Ar), 7.03 (s, 1H, Ar), 6.90 (d, *J* = 8.0 Hz, 1H, Ar), 4.83 (s, 2H, -CH_2_-); ^13^C NMR (DMSO-d_6_, 125 MHz) δ: 167.47 (C=O), 165.65 (C=O), 161.30 (oxazole C_2_), 158.44 (ArC-OH), 138.03 (oxazole C_4_), 136.52 (oxazole C_5_), 134.04 (-CH=), 134.55, 131.26, 130.94, 129.65, 127.04, 126.45, 121.90, 121.38, 118.54 (9 aromatic carbons), 116.52 (TZD C_5_=), 37.72 (-CH_2_-).

*(Z)-5-(3-Hydroxybenzylidene)-3-((2-(p-tolyl)oxazol-4-yl)methyl)thiazolidine-2,4-dione* (**5b**): C_21_H_16_N_2_O_4_S, calcd: C 64.27%, H 4.11%, N 7.14%, S 8.17%, found: C 64.31%, H 4.08%, N 7.19%, S 8.21%; white solid; mp = 229 °C; yield = 70%; FT IR (KBr) ν_max_ cm^−1^: 3432 (O-H), 3142 (C_5_-H oxazole), 1735 (C=O), 1680 (C=O), 1592 (C=N); MS: *m*/*z* = 393.2 (M + 1); ^1^H NMR (DMSO-d_6_, 500 MHz) δ: 10.01 (br, 1H, OH), 8.21 (s, 1H, oxazole C_5_-H), 7.89 (s, 1H, -CH=), 7.77 (d, *J* = 8.2 Hz, 2H, Ar), 7.35 (m, 3H, Ar), 7.10 (d, *J* = 7.7 Hz, 1H, Ar), 7.04 (s, 1H, Ar), 6.93 (d, *J* = 8.4 Hz, 1H, Ar), 4.84 (s, 2H, -CH_2_-), 2.58 (s, 3H, CH_3_-Ar); ^13^C NMR (DMSO-d_6_, 125 MHz) δ: 167.24 (C=O), 165.32 (C=O), 160.38 (oxazole C_2_), 158.31 (ArC-OH), 138.21 (oxazole C_4_), 135.87 (oxazole C_5_), 133.85 (-CH=), 141.15, 134.26, 131.25, 130.70, 126.99, 122.79, 122.03, 121.30, 118.45 (9 aromatic carbons), 116.52 (TZD C_5_=), 37.58 (-CH_2_-), 23.47 (-CH_3_).

*(Z)-3-((2-(4-Chlorophenyl)oxazol-4-yl)methyl)-5-(3-hydroxybenzylidene)thiazolidine-2,4-dione* (**5c**): C_20_H_13_ClN_2_O_4_S, calcd: C 58.18%, H 3.17%, N 6.79%, S 7.77%, found: C 58.25%, H 3.29%, N 6.70%, S 7.76%; white solid; mp = 227 °C; yield = 73%; FT IR (KBr) ν_max_ cm^−1^: 3407 (O-H), 3159 (C_5_-H oxazole), 1736 (C=O), 1684 (C=O), 1592 (C=N); MS: *m*/*z* = 413.4 and 415.5 (M + 1; ^35^Cl and ^37^Cl approx. 3:1 ratio); ^1^H NMR (DMSO-d_6_, 500 MHz) δ: 9.93 (br, 1H, OH), 8.23 (s, 1H, oxazole C_5_-H), 7.93 (d, *J* = 8.7 Hz, 2H, Ar), 7.87 (s, 1H, -CH=), 7.51 (d, *J* = 8.7 Hz, 2H, Ar), 7.37 (t, *J* = 8.4 Hz, 1H, Ar), 7.09 (d, *J* = 8.4 Hz, 1H, Ar), 7.02 (s, 1H, Ar), 6.92 (d, *J* = 8.4 Hz, 1H, Ar), 4.81 (s, 2H, -CH_2_-); ^13^C NMR (DMSO-d_6_, 125 MHz) δ: 167.51 (C=O), 165.26 (C=O), 160.31 (oxazole C_2_), 158.21 (ArC-OH), 138.20 (oxazole C_4_), 135.99 (oxazole C_5_), 133.91 (-CH=), 137.21, 134.87, 131.17, 129.69, 128.35, 124.61, 121.85, 121.18, 118.37 (9 aromatic carbons), 116.56 (TZD C_5_=), 37.68 (-CH_2_-).

*(Z)-5-(3-Hydroxybenzylidene)-3-((2-(4-nitrophenyl)oxazol-4-yl)methyl)thiazolidine-2,4-dione* (**5d**): C_20_H_13_N_3_O_6_S, calcd: C 56.73%, H 3.09%, N 9.92%, S 7.57%, found: C 56.73%, H 3.08%, N 9.91%, S 7.58%; yellow solid; mp = 255 °C; yield = 72%; FT IR (KBr) ν_max_ cm^−1^: 3369 (O-H), 3164 (C_5_-H oxazole), 1731 (C=O), 1669 (C=O), 1593 (C=N), 1515, 1342 (N=O); MS: *m*/*z* = 424.3 (M + 1); ^1^H NMR (DMSO-d_6_, 500 MHz) δ: 9.95 (br, 1H, OH), 8.24 (s, 1H, oxazole C_5_-H), 8.15 (d, *J* = 8.6 Hz, 2H, Ar), 8.09 (d, *J* = 8.6 Hz, 2H, Ar), 7.88 (s, 1H, -CH=), 7.38 (t, *J* = 8.2 Hz, 1H, Ar), 7.09 (d, *J* = 8.1 Hz, 1H, Ar), 7.02 (s, 1H, Ar), 6.91 (d, *J* = 8.1 Hz, 1H, Ar), 4.83 (s, 2H, -CH_2_-); ^13^C NMR (DMSO-d_6_, 125 MHz) δ: 167.29 (C=O), 165.24 (C=O), 160.29 (oxazole C_2_), 158.56 (ArC-OH), 138.29 (oxazole C_4_), 136.09 (oxazole C_5_), 134.26 (-CH=), 146.01, 134.51, 131.06, 128.61, 126.65, 124.29, 122.01, 121.35, 118.61 (9 aromatic carbons), 116.33 (TZD C_5_=), 37.60 (-CH_2_-).

*(Z)-5-(2-Hydroxybenzylidene)-3-((2-phenyloxazol-4-yl)methyl)thiazolidine-2,4-dione* (6**a**): C_20_H_14_N_2_O_4_S, calcd: C 63.48%, H 3.73%, N 7.40%, S 8.47%, found: C 63.46%, H 3.75%, N 7.42%, S 8.44%; yellow solid; mp = 217 °C; yield = 40%; FT IR (KBr) ν_max_ cm^−1^: 3409 (O-H), 3152 (C_5_-H oxazole), 1731 (C=O), 1668 (C=O), 1596 (C=N); MS: *m*/*z* = 379.1 (M + 1); ^1^H NMR (DMSO-d_6_, 500 MHz) δ: 10.59 (br, 1H, OH), 8.24 (s, 1H, oxazole C_5_-H), 8.12 (s, 1H, -CH=), 7.94 (d, *J* = 8.0 Hz, 2H, Ar), 7.54–51 (m, 3H, Ar), 7.38–7.34 (m, 2H, Ar), 6.97–6.96 (m, 2H, Ar), 4.83 (s, 2H, -CH_2_-); ^13^C NMR (DMSO-d_6_, 125 MHz) δ: 167.72 (C=O), 165.87 (C=O), 161.28 (oxazole C_2_), 157.85 (ArC-OH), 138.00 (oxazole C_4_), 136.60 (oxazole C_5_), 133.09 (-CH=), 131.25, 129.64, 129.16, 129.11, 126.45, 127.05, 120.31, 120.07, 120.05 (9 aromatic carbons), 116.71 (TZD C_5_=), 37.62 (-CH_2_-).

*(Z)-5-(2-Hydroxybenzylidene)-3-((2-(p-tolyl)oxazol-4-yl)methyl)thiazolidine-2,4-dione* (**6b**): C_21_H_16_N_2_O_4_S, calcd: C 64.27%, H 4.11%, N 7.14%, S 8.17%, found: C 64.38%, H 4.18%, N 7.15%, S 8.28%; yellow solid; mp = 205 °C; yield = 38%; FT IR (KBr) ν_max_ cm^−1^: 3432 (O-H), 3151 (C_5_-H oxazole), 1730 (C=O), 1686 (C=O), 1590 (C=N); MS: *m*/*z* = 393.2 (M + 1); ^1^H NMR (DMSO-d_6_, 500 MHz) δ: 10.28 (br, 1H, OH), 8.30 (s, 1H, oxazole C_5_-H), 8.05 (s, 1H, -CH=), 7.68 (d, *J* = 8.2 Hz, 2H, Ar), 7.31–7.35 (m, 4H, Ar), 6.96 (m, 2H, Ar), 4.79 (s, 2H, -CH_2_-), 2.56 (s, 3H, CH_3_-Ar); ^13^C NMR (DMSO-d_6_, 125 MHz) δ: 167.11 (C=O), 165.06 (C=O), 160.17 (oxazole C_2_), 157.32 (ArC-OH), 138.19 (oxazole C_4_), 135.89 (oxazole C_5_), 132.84 (-CH=), 141.29, 130.71, 129.05, 128.99, 126.01, 122.68, 120.87, 120.64, 120.36 (9 aromatic carbons), 116.93 (TZD C_5_=), 37.61 (-CH_2_-), 23.52 (-CH_3_).

*(Z)-3-((2-(4-Chlorophenyl)oxazol-4-yl)methyl)-5-(2-hydroxybenzylidene)thiazolidine-2,4-dione* (**6c**): C_20_H_13_ClN_2_O_4_S, calcd: C 58.18%, H 3.17%, N 6.79%, S 7.77%, found: C 58.17%, H 3.16%, N 6.76%, S 7.74%; yellow solid; mp = 257 °C; yield = 41%; FT IR (KBr) ν_max_ cm^−1^: 3413 (O-H), 3157 (C_5_-H oxazole), 1731 (C=O), 1668 (C=O), 1595 (C=N); MS: *m*/*z* = 413.4 and 415.2 (M + 1; ^35^Cl and ^37^Cl approx. 3:1 ratio); ^1^H NMR (DMSO-d_6_, 500 MHz) δ: 10.61 (br, 1H, OH), 8.25 (s, 1H, oxazole C_5_-H), 8.18 (s, 1H, -CH=), 7.93 (d, *J* = 8.5 Hz, 2H, Ar), 7.50 (d, *J* = 8.5 Hz, 2H, Ar), 7.37–7.35 (m, 2H, Ar), 6.97–6.99 (m, 2H, Ar), 4.83 (s, 2H, -CH_2_-); ^13^C NMR (DMSO-d_6_, 125 MHz) δ: 167.41 (C=O), 165.87 (C=O), 160.54 (oxazole C_2_), 158.09 (ArC-OH), 138.27 (oxazole C_4_), 135.81 (oxazole C_5_), 132.95 (-CH=), 137.09, 129.71, 129.44, 129.14, 128.37, 124.15, 120.99, 120.90, 120.23 (9 aromatic carbons), 116.77 (TZD C_5_=), 37.72 (-CH_2_-).

*(Z)-5-(2-Hydroxybenzylidene)-3-((2-(4-nitrophenyl)oxazol-4-yl)methyl)thiazolidine-2,4-dione* (**6d**): C_20_H_13_N_3_O_6_S, calcd: C 56.73%, H 3.09%, N 9.92%, S 7.57%, found: C 56.76%, H 3.11%, N 9.88%, S 7.60%; yellow solid; mp = 255–256 °C; yield = 40%; FT IR (KBr) ν_max_ cm^−1^: 3503 (O-H), 3165 (C_5_-H oxazole), 1718 (C=O), 1665 (C=O), 1600 (C=N), 1520, 1336 (N=O); MS: *m*/*z* = 424.6 (M + 1); ^1^H NMR (DMSO-d_6_, 500 MHz) δ: 10.67 (br, 1H, OH), 8.25 (s, 1H, oxazole C_5_-H), 8.17 (s, 1H, -CH=), 8.10 (d, *J* = 8.3 Hz, 2H, Ar), 8.06 (d, *J* = 8.3 Hz, 2H, Ar), 7.37–7.35 (m, 2H, Ar), 6.98 (m, 2H, Ar), 4.81 (s, 2H, -CH_2_-); ^13^C NMR (DMSO-d_6_, 125 MHz) δ: 167.81 (C=O), 165.31 (C=O), 160.01 (oxazole C_2_), 157.03 (ArC-OH), 138.35 (oxazole C_4_), 135.98 (oxazole C_5_), 132.25 (-CH=), 145.84, 129.13, 128.67, 128.57, 126.66, 124.15, 121.44, 121.09, 120.11 (9 aromatic carbons), 117.03 (TZD C_5_=), 37.54 (-CH_2_-).

*(Z)-5-(4-Hydroxy-3-methoxybenzylidene)-3-((2-phenyloxazol-4-yl)methyl)thiazolidine-2,4-dione* (**7a**): C_21_H_16_N_2_O_5_S, calcd: C 61.76%, H 3.95%, N 6.86%, S 7.85%, found: C 61.76%, H 3.92%, N 6.87%, S 7.88%; yellow solid; mp = 190 °C; yield = 63%; FT IR (KBr) ν_max_ cm^−1^: 3523 (O-H), 3140 (C_5_-H oxazole), 1733 (C=O), 1684 (C=O), 1594 (C=N), 1284 (C-O-C); MS: *m*/*z* = 409.3 (M + 1); ^1^H NMR (DMSO-d_6_, 500 MHz) δ: 10.26 (br, 1H, OH), 8.24 (s, 1H, oxazole C_5_-H), 7.93 (d, *J* = 7.8 Hz, 2H, Ar), 7.85 (s, 1H, -CH=), 7.65 (s, 1H, Ar), 7.60 (d, *J* = 8.2 Hz, 1H, Ar), 7.54–7.52 (m, 3H, Ar), 6.92 (d, *J* = 8.2 Hz, 1H, Ar), 4.82 (s, 2H, -CH_2_-), 3.73 (s, 3H, -O-CH_3_); ^13^C NMR (DMSO-d_6_, 125 MHz) δ: 167.59 (C=O), 165.13 (C=O), 161.30 (oxazole C_2_), 156.09 (ArC-OCH_3_), 155.27 (ArC-OH), 137.98 (oxazole C_4_), 136.61 (oxazole C_5_), 133.17 (-CH=), 133.01, 131.09, 130.08, 129.61, 128.21, 127.03, 126.43, 125.72 (8 aromatic carbons), 116.36 (TZD C_5_=), 55.89 (-CH_3_), 37.55 (-CH_2_-).

*(Z)-5-(4-Hydroxy-3-methoxybenzylidene)-3-((2-(p-tolyl)oxazol-4-yl)methyl)thiazolidine-2,4-dione* (**7b**): C_22_H_18_N_2_O_5_S, calcd: C 62.55%, H 4.29%, N 6.63%, S 7.59%, found: C 62.60%, H 4.35%, N 7.58%, S 8.51%; yellow solid; mp = 205 °C; yield = 57%; FT IR (KBr) ν_max_ cm^−1^: 3412 (O-H), 3132 (C_5_-H oxazole), 1742 (C=O), 1693 (C=O), 1590 (C=N), 1266 (C-O-C); MS: *m*/*z* = 423.2 (M + 1); ^1^H NMR (DMSO-d_6_, 500 MHz) δ: 10.32 (br, 1H, OH), 8.26 (s, 1H, oxazole C_5_-H), 7.89 (s, 1H, -CH=), 7.69 (d, *J* = 8.1 Hz, 2H, Ar), 7.61–7.63 (m, 2H, Ar), 7.27 (d, *J* = 8.1 Hz, 2H, Ar), 6.94 (d, *J* = 7.9 Hz, 1H, Ar), 4.77 (s, 2H, -CH_2_-), 3.78 (s, 3H, -O-CH_3_), 2.64 (s, 3H, CH_3_-Ar); ^13^C NMR (DMSO-d_6_, 125 MHz) δ: 167.34 (C=O), 165.16 (C=O), 160.81 (oxazole C_2_), 155.91 (ArC-OCH_3_), 155.62 (ArC-OH), 138.28 (oxazole C_4_), 135.79 (oxazole C_5_), 133.67 (-CH=), 141.80, 134.29, 130.71, 129.21, 128.26, 126.81, 125.89, 124.19 (8 aromatic carbons), 116.19 (TZD C_5_=), 55.34 (-CH_3_), 37.61 (-CH_2_-), 23.51 (-CH_3_).

*(Z)-3-((2-(4-Chlorophenyl)oxazol-4-yl)methyl)-5-(4-hydroxy-3-methoxybenzylidene)thiazolidine-2,4-dione* (**7c**): C_21_H_15_ClN_2_O_5_S, calcd: C 59.95%, H 3.41%, N 6.33%, S 7.24%, found: C 59.95%, H 3.43%, N 6.35%, S 7.20%; orange-yellow solid; mp = 247 °C; yield = 63%; FT IR (KBr) ν_max_ cm^−1^: 3421 (O-H), 3149 (C_5_-H oxazole), 1727 (C=O), 1670 (C=O), 1589 (C=N), 1239 (C-O-C); MS: *m*/*z* = 443.2 and 445.6 (M + 1; ^35^Cl and ^37^Cl approx. 3:1 ratio); ^1^H NMR (DMSO-d_6_, 500 MHz) δ: 10.20 (br, 1H, OH), 8.24 (s, 1H, oxazole C_5_-H), 7.94 (d, *J* = 8.4 Hz, 2H, Ar), 7.89 (s, 1H, -CH=), 7.64–7.61 (m, 2H, Ar), 7.50 (d, *J* = 8.4 Hz, 2H, Ar), 6.93 (d, *J* = 7.9 Hz, 1H, Ar), 4.81 (s, 2H, -CH_2_-), 3.72 (s, 3H, -O-CH_3_); ^13^C NMR (DMSO-d_6_, 125 MHz) δ: 167.91 (C=O), 165.83 (C=O), 160.61 (oxazole C_2_), 156.01 (ArC-OCH_3_), 155.19 (ArC-OH), 138.31 (oxazole C_4_), 135.99 (oxazole C_5_), 132.88 (-CH=), 137.18, 132.80, 129.45, 129.16, 128.44, 128.19, 125.60, 124.53 (8 aromatic carbons), 116.91 (TZD C_5_=), 55.60 (-CH_3_), 37.59 (-CH_2_-).

*(Z)-5-(4-Hydroxy-3-methoxybenzylidene)-3-((2-(4-nitrophenyl)oxazol-4-yl)methyl)thiazolidine-2,4-dione* (**7d**): C_21_H_15_N_3_O_7_S, calcd: C 55.63%, H 3.33%, N 9.27%, S 7.07%, found: C 55.59%, H 3.32%, N 9.28%, S 7.08%; yellow solid; mp = 233–234 °C; yield = 75%; FT IR (KBr) ν_max_ cm^−1^: 3398 (O-H), 3106 (C_5_-H oxazole), 1739 (C=O), 1669 (C=O), 1588 (C=N), 1512, 1341 (N=O), 1271 (C-O-C); MS: *m*/*z* = 454.5 (M + 1); ^1^H NMR (DMSO-d_6_, 500 MHz) δ: 10.35 (br, 1H, OH), 8.23 (s, 1H, oxazole C_5_-H), 8.14 (d, *J* = 8.4 Hz, 2H, Ar), 8.07 (d, *J* = 8.4 Hz, 2H, Ar), 7.88 (s, 1H, -CH=), 7.62 (s, 1H, Ar), 7.57 (d, *J* = 8.0 Hz, 1H, Ar), 6.95 (d, *J* = 8.0 Hz, 1H, Ar), 4.81 (s, 2H, -CH_2_-), 3.70 (s, 3H, -O-CH_3_); ^13^C NMR (DMSO-d_6_, 125 MHz) δ: 167.41 (C=O), 165.09 (C=O), 160.54 (oxazole C_2_), 155.74 (ArC-OCH_3_), 155.38 (ArC-OH), 138.37 (oxazole C_4_), 136.07 (oxazole C_5_), 133.80 (-CH=), 145.77, 135.13, 129.17, 128.49, 128.38, 126.39, 125.31, 124.99 (8 aromatic carbons), 116.52 (TZD C_5_=), 55.12 (-CH_3_), 37.66 (-CH_2_-).

### 3.3. Biological Assays

The biologic activity of the final compounds **4a**–**d**, **5a**–**d**, **6a**–**d** and **7a**–**d** was assessed by using 3 distinct approaches. The antimicrobial potential was determined for all compounds via an initial in vitro qualitative screening study, followed by an in vitro quantitative assay. Furthermore, we investigated the anti-biofilm, and thus antipathogenic potential of the new compounds.

Our aim was to investigate the specificity of the compounds in their predicted biologic activity as anti-Candida biofilm agents. In order to prove the lack of direct antibacterial or antifungal effect, we selected an array of 2 Gram-positive strains (*Staphylococcus aureus* ATCC 25923, *Enterococcus faecalis* ATCC 29212), 2 Gram-negative strains (*Pseudomonas aeruginosa* ATCC 27853, *Escherichia coli* ATCC 25922) and 1 fungal strain (*Candida albicans* ATCC 10231). These strains were reference strains and their identity was confirmed using the VITEK 1 automatic system. Anti-biofilm tests were performed for all compounds investigated, regardless of their activity as direct antimicrobial agents, as it was this paper’s aim to obtain and to prove that the new molecules selectively inhibit *Candida* biofilm formation and do not affect other microbial biofilm, nor do they have direct antimicrobial action.

#### 3.3.1. Antimicrobial Activity—Initial In Vitro Qualitative Screening Study

This initial screening was performed using an adapted disk diffusion technique, previously reported [49,59,60,61]. All tested compounds and standards were solubilized in dimethylsulfoxide (DMSO) to a concentration of 1 mg/mL. Microbial inocula (saline suspension of 0.5 McFarland density), obtained from microbial cultures grown on solid media for 15–18 h, were seeded on solid Muller-Hinton medium. The solutions where then applied directly on the solid medium and the resulting plates were allowed to incubate for 24 h at 37 °C and 48 h at 28 °C for the fungal strain. Antimicrobial activity was assessed as the diameter of the growth inhibition area, measured in mm.

#### 3.3.2. Antimicrobial Activity—In Vitro Quantitative Assay

The quantitative assay was performed using 96-wells plates containing liquid Mueller-Hinton medium seeded with 20 μL microbial inoculum. The stock solutions of the tested compounds were prepared at concentrations of 5 mg/mL in DMSO. They were applied as two-fold serial dilutions ranging from 2500 µg to 2 µg mL^−1^. The total broth volume was adjusted to 200 µg mL^−1^. Standard antimicrobial agents were used (norfloxacin, fluconazole). Culture positive controls and blank DMSO dilution were used. The plates were incubated for 24 h at 37 °C for bacterial strains and 48 h at 28 °C for the fungal strain. The minimal inhibitory concentration (MIC) values were determined as the lowest concentration of the investigated compound that inhibited the growth of the microbial cultures, compared to the positive control, as established by a decreased value of absorbance at 600 nm (Apollo LB 911 ELISA Absorbance Reader, Berthold Technologies, Bad Wildbad, Germany) [60,62,63,64].

#### 3.3.3. Anti-Biofilm Activity Assay

The microtiter plate method, previously reported [60,65], was used to ascertain the level of anti-biofilm activity of the tested compounds. In order to determine the ability to colonize inert substratum, the plates previously used for MIC determination were emptied, rinsed 3 times with phosphate buffered saline and then fixed with cold methanol 80% for 5 min. The biofilm was stained with violet crystal for 30 min, and then washed multiple times with water and finally suspended using a glacial acetic acid solution. Cell density was measured by evaluating the optical density of the colored solution at 490 nm. The lowest concentration of the compounds that inhibited the development of biofilm on the plate wells was considered the minimal biofilm eradication concentration (MBEC).

### 3.4. Molecular Docking Study

The tested compounds were docked in the binding site of the most important adhesion proteins of *C. albicans*, in order to understand the differences between compounds in terms of interaction with the microorganism’s adhesion proteins. Our in silico study focused on finding differences of interactions between our compounds and the target proteins from the point of view of the various substitutions and isomerism in our molecules and from the point of view of the differences between the macromolecular targets.

For Als3 and Als9, 3D structures obtained by X-ray diffraction were deposited in Protein Data Bank (PDB), as presented in Table 9. Because no structure could be found for the other members of the Candida Als superfamily, they were built by homology modeling. For this purpose, FASTA primary sequences of amino acid for the target proteins were taken from UniProt [66]. Using Swiss-Model [67], the new structures were generated, based on proposed PDB template structures with high coverage and identity percent with the primary amino acid sequence. The FASTA amino acid sequences used, the structures used as templates, and the degree of identity are presented in Table 10.

The files of ligands (final compounds **4a**–**d**, **5a**–**d**, **6a**–**d** and **7a**–**d**) and the macromolecular targets were prepared as reported [60], using AutoDock Tools 1.5.6 [68]. In all structures the polar hydrogens were added, the non-polar hydrogens were merged and the partial charges were added. Amide bonds were configured as rigid.

The Cartesian coordinates of the search space center for all Als proteins are presented in Table 9 and Table 10. The search space was defined as x = y = z = 74 Å for all targets, in order to provide equal experimental conditions for all interaction predictions. The search space center Cartesian coordinates was configured in order to fit the entire active pocket of all Als surface proteins.

The molecular docking study was performed using AutoDock 4.2 [68], 30 conformations were searched for every ligand-protein complex. The inhibition constant (Ki) was calculated based on the computed binding affinity energy (∆G) using the formula: $Ki=e^{\frac{\Delta G\times 1000}{R\times T}}$, where R represents the Regnault constant = 198,719 and T = 298.15 K.

Alignment of the primary structure and the similarity of the tested Als proteins was performed using Clustal Omega [69]. Analysis of the binding pocket of the Als proteins was performed using DoGSiteScorer [70,71].

## 4. Conclusions

In an effort to obtain new agents that target *C. albicans* biofilm development, following an extensive review of the literature, we proposed a new molecular scaffold: *N*-(oxazolylmethyl)-thiazolidindione. A series of 16 new compounds bearing this moiety were synthesized and their structures were confirmed using physicochemical parameters and spectral data.

A general antimicrobial activity screening was performed using both qualitative and quantitative methods against Gram-positive and Gram-negative bacteria, as well as fungi. Results showed that the compounds do not possess significant direct antimicrobial activity and thus are not estimated to determine selection pressure or to affect non-pathogenic commensal flora.

The biologic anti-biofilm evaluation demonstrated that, as hypothesized when constructing this scaffold, the compounds are very active selectively against *C. albicans* biofilm formation. In order to provide a possible mechanism of action, we performed a docking study that proved these compounds have a very good binding potential against most of the Als surface proteins of *C. albicans.* All compounds seem to be able to bind to Als1, Als5 and Als6, while some are also capable of good interactions with Als3. Considering the well documented role of Als1, Als3 and Als5 as adhesins and key agents in biofilm formation, we postulate that these compounds selectively inhibit *C. albicans* biofilm formation most likely by interfering with the Als proteins.

## Figures and Tables

**Table 1:** The antimicrobial activity of the tested compounds expressed as zone diameters of microbial growth inhibition (mm).

Compound	*S. aureus*	*E. faecalis*	*P. aeruginosa*	*E. coli*	*C. albicans*
	ATCC 5923	ATCC 29212	ATCC 27853	ATCC 25922	ATCC 10231
**4a**	10	12	12	8	16
**4b**	8	12	12	12	14
**4c**	8	12	12	12	16
**4d**	8	16	12	12	14
**5a**	10	12	10	12	14
**5b**	10	12	12	12	12
**5c**	10	12	12	10	14
**5d**	8	15	10	12	12
**6a**	10	12	8	10	16
**6b**	8	12	10	12	12
**6c**	10	12	12	12	14
**6d**	16	15	10	12	14
**7a**	10	12	8	12	16
**7b**	10	12	8	10	12
**7c**	6	15	12	16	14
**7d**	16	15	12	16	14
**Fluconazole**	-	-	-	-	24
**Norfloxacin**	26	26	26	26	-
**DMSO**	0	0	0	0	0

**Table 2:** The minimum inhibitory concentrations MIC (mg mL^−1^) values of the new compounds against the tested microbial strains.

Compound	*S. aureus*	*E. faecalis*	*P. aeruginosa*	*E. coli*	*C. albicans*
	ATCC 25923	ATCC 29212	ATCC 27853	ATCC 25922	ATCC 10231
**4a**	2.5	2.5	2.5	2.5	0.625
**4b**	2.5	2.5	2.5	2.5	0.625
**4c**	2.5	2.5	2.5	1.25	0.625
**4d**	2.5	2.5	2.5	2.5	0.625
**5a**	2.5	2.5	2.5	2.5	0.625
**5b**	2.5	2.5	2.5	2.5	0.625
**5c**	2.5	2.5	2.5	1.25	0.625
**5d**	2.5	2.5	2.5	2.5	0.625
**6a**	2.5	2.5	2.5	2.5	0.625
**6b**	2.5	2.5	2.5	1.25	0.625
**6c**	2.5	2.5	2.5	2.5	0.625
**6d**	2.5	2.5	2.5	2.5	0.625
**7a**	2.5	2.5	2.5	1.25	0.625
**7b**	2.5	2.5	2.5	2.5	0.625
**7c**	2.5	2.5	2.5	0.625	0.625
**7d**	2.5	2.5	2.5	0.625	0.625
**Fluconazole**	-	-	-	-	0.004
**Norfloxacin**	0.002	0.004	0.002	0.002	-

**Table 3:** The minimal biofilm eradication concentration MBEC (mg mL^−1^) values of the final compounds against various microbial strains.

Compound	*S. aureus*	*E. faecalis*	*P. aeruginosa*	*E. coli*	*C. albicans*
	ATCC 25923	ATCC 29212	ATCC 27853	ATCC 25922	ATCC 10231
**4a**	>0.625	>0.625	0.625	0.625	0.078
**4b**	0.625	0.625	0.625	0.625	0.078
**4c**	0.625	0.312	0.625	0.312	0.078
**4d**	0.625	0.625	0.625	0.625	0.078
**5a**	0.312	0.312	0.625	0.625	0.078
**5b**	>0.625	>0.625	0.625	0.625	0.078
**5c**	>0.625	>0.625	0.625	0.625	0.078
**5d**	0.625	>0.625	0.625	0.625	0.039
**6a**	0.625	0.625	0.625	0.625	0.078
**6b**	>0.625	0.156	0.625	0.312	0.156
**6c**	0.625	0.156	0.625	0.625	0.156
**6d**	>0.625	>0.625	0.625	0.625	>0.625
**7a**	>0.625	>0.625	>0.625	>0.625	>0.625
**7b**	>0.625	0.625	0.625	0.625	0.078
**7c**	>0.625	0.625	0.625	0.312	0.078
**7d**	0.625	0.156	0.625	0.312	0.156
**Berberine**	0.078	0.156	0.625	0.625	0.312

**Table 4:** Binding energies (kcal/mol) of the tested compounds-Als complexes.

Compound	*Candida albicans* Als Surface Proteins							
	Als1	Als2	Als3	Als4	Als5	Als6	Als7	Als9
**4a**	−11.21	−9.65	−10.55	−9.34	−11.26	−11.33	−10.37	−10.30
**4b**	−11.43	−9.91	−10.81	−9.67	−11.41	−11.58	−11.02	−10.65
**4c**	−11.14	−9.61	−10.37	−9.76	−11.68	−11.64	−10.91	−10.55
**4d**	−11.05	−9.82	−10.08	−9.35	−11.73	−11.64	−10.83	−10.12
**5a**	−11.57	−10.16	−10.14	−9.62	−11.35	−11.21	−10.09	−9.97
**5b**	−11.83	−9.68	−10.74	−9.69	−11.73	−11.76	−10.99	−10.53
**5c**	−11.51	−9.52	−10.60	−9.77	−11.69	−11.74	−11.19	−10.36
**5d**	−11.98	−10.05	−10.81	−9.82	−11.52	−11.82	−11.95	−10.54
**6a**	−10.98	−9.58	−10.47	−9.22	−11.21	−11.36	−10.19	−9.65
**6b**	−10.87	−9.82	−10.47	−9.69	−11.70	−11.17	−10.83	−9.77
**6c**	−10.99	−9.79	−10.43	−9.52	−11.20	−11.02	−10.39	−9.73
**6d**	−10.43	−9.23	−9.55	−9.02	−10.99	−11.09	−9.99	−9.18
**7a**	−11.05	−8.99	−10.05	−9.31	−10.78	−11.11	−9.90	−10.01
**7b**	−11.61	−9.26	−10.30	−9.77	−11.47	−11.60	−10.25	−10.17
**7c**	−11.12	−9.49	−10.28	−9.55	−11.66	−11.34	−10.50	−10.55
**7d**	−10.99	−9.25	−9.16	−9.42	−11.03	−11.30	−11.24	−9.26
**Berberine**	−9.85	−8.03	−8.26	−7.97	−7.87	−8.57	−7.85	−9.32

**Table 5:** Binding affinity average comparison between different structural subseries.

Compounds	Series Type	Binding Affinity (kcal/mol)	
		Average	Standard Deviation
**4a, 5a, 6a, 7a**	2-phenyloxazole	−10.37	0.147
**4b, 5b, 6b, 7b**	2-(p-tolyl)oxazole	−10.69	
**4c, 5c, 6c, 7c**	2-(4-chlorophenyl)oxazole	−10.61	
**4d, 5d, 6d, 7d**	2-(4-nitrophenyl)oxazole	−10.45	
**4a–d**	4-hydroxy-phenyl	−10.65	0.240
**5a–d**	3-hydroxy-phenyl	−10.81	
**6a–d**	2-hydroxy-phenyl	−10.30	
**7a–d**	4-hydroxy-3-methoxybenzylidene	−10.37	

**Table 6:** The predicted inhibition constants Ki (nM) for the tested compounds-Als complexes.

Compound	Als1	Als2	Als3	Als4	Als5	Als6	Als7	Als9
**4a**	6.07	84.42	18.48	142.46	5.58	4.95	25.04	28.18
**4b**	4.18	54.43	11.92	81.62	4.33	3.25	8.36	15.61
**4c**	6.83	90.32	25.04	70.12	2.74	2.94	10.07	18.48
**4d**	7.95	63.36	40.86	140.07	2.52	2.94	11.52	38.19
**5a**	3.30	35.70	36.92	88.81	4.79	6.07	40.17	49.19
**5b**	2.13	80.25	13.41	78.91	2.52	2.40	8.79	19.12
**5c**	3.66	105.13	16.99	68.94	2.70	2.48	6.28	25.47
**5d**	1.65	42.98	11.92	63.36	3.60	2.17	1.74	18.80
**6a**	8.94	95.01	21.15	174.44	6.07	4.71	33.93	84.42
**6b**	10.77	63.36	21.15	78.91	2.65	6.49	11.52	68.94
**6c**	8.79	66.65	22.63	105.13	6.17	8.36	24.21	73.76
**6d**	22.63	171.52	99.94	244.48	8.79	7.43	47.56	186.62
**7a**	7.95	257.18	42.98	149.86	12.54	7.18	55.36	45.98
**7b**	3.09	163.05	28.18	68.94	3.91	3.14	30.67	35.10
**7c**	7.06	110.59	29.15	99.94	2.84	4.87	20.11	18.48
**7d**	8.79	165.83	193.03	124.46	8.22	5.21	5.77	163.05
**Berberine**	60.23	1299.95	881.73	1438.49	1702.98	522.52	1761.44	147.35

**Table 7:** The similarity matrix of the primary structures of different Als.

	Als1	Als2	Als3	Als4	Als5	Als6	Als7	Als9
**Als1**	100.00	74.16	82.61	62.54	81.27	64.55	46.58	68.46
**Als2**	74.16	100.00	74.16	60.54	73.46	61.76	45.36	64.94
**Als3**	82.61	74.16	100.00	63.21	77.59	63.55	47.95	68.56
**Als4**	62.54	60.54	63.21	100.00	58.33	56.67	44.18	64.21
**Als5**	81.27	73.46	77.59	58.33	100.00	61.24	43.49	65.92
**Als6**	64.55	61.76	63.55	56.67	61.24	100.00	50.00	61.76
**Als7**	46.58	45.36	47.95	44.18	43.49	50.00	100.00	46.39
**Als9**	68.46	64.94	68.56	64.21	65.92	61.76	46.39	100.00

**Table 8:** The Als’ binding pocket characteristics.

Parameter	Als1	Als2	Als3	Als4	Als5	Als6	Als7	Als9
**Volume (Å^3^)**	1510.21	1382.53	1496.96	1447.10	1640.26	1142.21	1218.05	1488.19
**Internal surface (Å^2^)**	1766.14	1551.69	1603.54	1539.99	1963.5	1168.52	1583.70	1473.79
**H bond donors**	39	40	36	31	43	30	33	29
**H bond acceptors**	107	94	111	86	131	61	92	102
**Hydrophobic residues**	74	62	61	70	82	56	95	79
**Hydrophobicity ratio**	34%	31%	29%	37%	32%	38%	43%	38%
**Apolar AA ratio**	36%	36%	41%	41%	35%	42%	35%	42%
**Polar AA ratio**	52%	45%	48%	45%	51%	46%	42%	38%
**Cationic AA ratio**	6%	9%	5%	7%	9%	8%	10%	6%
**Anionic AA ratio**	6%	9%	5%	7%	5%	4%	13%	14%

**Table 9:** Target macromolecular structures retrieved from Protein Data Bank (PDB).

Target	PDB Entry Code	Cartesian Coordinates of the Search Space Center		
		x	y	Z
Als3	4LEE	21.248	0.862	51.807
Als9	2YLH	−7.88	24.311	−10.076

**Table 10:** Structures built by homology modeling (not available from Protein Data Bank).

Target	FASTA Amino Acid Sequence	PDB Entry Template	Identity (%)	Cartesian Coordinates of the Search Space Center		
				x	y	z
Als1	Q5A874	4LEE	82.43	21.248	0.862	51.807
Als2	Q9URQ0	4LEE	73.46	21.248	0.862	51.807
Als4	O7466Q	2Y7M	65.03	30.540	53.025	12.815
Als5	Q5A8T7	4LEE	77.64	21.248	0.862	51.807
Als6	Q5A2Z7	2YLH	62.42	−7.88	24.311	−10.076
Als7	Q5A312	4LEE	47.26	21.248	0.862	51.807
